# Dihydromyricetin increases endothelial nitric oxide production and inhibits atherosclerosis through microRNA‐21 in apolipoprotein E‐deficient mice

**DOI:** 10.1111/jcmm.15278

**Published:** 2020-04-17

**Authors:** Dafeng Yang, Zhousheng Yang, Lei Chen, Dabin Kuang, Yang Zou, Jie Li, Xu Deng, Songyuan Luo, Jianfang Luo, Jun He, Miao Yan, Guixia He, Yang Deng, Rong Li, Qiong Yuan, Yangzhao Zhou, Pei Jiang, Shenglan Tan

**Affiliations:** ^1^ Department of Pharmacy Institute of Clinical Pharmacy Second Xiangya Hospital Central South University Changsha China; ^2^ Department of Cardiovascular Surgery Second Xiangya Hospital Central South University Changsha China; ^3^ Department of Pharmacy The People’s Hospital of Guangxi Zhuang Autonomous Region Nanning China; ^4^ Department of Pharmacy Affiliated Changsha Hospital of Hunan Normal University Changsha China; ^5^ Department of Geriatrics National Key Clinic Specialty Guangzhou First People’s Hospital Guangzhou Medical University Guangzhou China; ^6^ Department of Cardiology Third Xiangya Hospital Central South University Changsha China; ^7^ Department of Cardiology Vascular Center Guangdong Cardiovascular Institute Guangdong Provincial Key Laboratory of Coronary Heart Disease Prevention Guangdong Provincial People’s Hospital Guangdong Academy of Medical Sciences Guangzhou China; ^8^ Department of General Surgey Second Xiangya Hospital Central South University Changsha China; ^9^ School of Pharmacy Hunan University of Chinese Medicine Changsha China; ^10^ Department of Pharmacy The Third Hospital of Changsha Changsha China; ^11^ The Second Affiliated Hospital of University of South China Hengyang China; ^12^ New Drugs Innovation and Development Institute Department of Pharmacy College of Medicine Wuhan University of Science and Technology Wuhan China; ^13^ Department of Clinical Pharmacy and Pharmacology Jining First People’s Hospital Jining Medical University Jining China

**Keywords:** atherosclerosis, dihydromyricetin, endothelial cell, microRNA, nitric oxide

## Abstract

Natural products were extracted from traditional Chinese herbal emerging as potential therapeutic drugs for treating cardiovascular diseases. This study examines the role and underlying mechanism of dihydromyricetin (DMY), a natural compound extracted from *Ampelopsis grossedentata*, in atherosclerosis. DMY treatment significantly inhibits atherosclerotic lesion formation, proinflammatory gene expression and the influx of lesional macrophages and CD4‐positive T cells in the vessel wall and hepatic inflammation, whereas increases nitric oxide (NO) production and improves lipid metabolism in apolipoprotein E‐deficient (*Apoe^−^* ^/^ *^−^*) mice. Yet, those protective effects are abrogated by using NOS inhibitor L‐NAME in *Apoe^−^* ^/^ *^−^* mice received DMY. Mechanistically, DMY decreases microRNA‐21 (miR‐21) and increases its target gene dimethylarginine dimethylaminohydrolase‐1 (DDAH1) expression, an effect that reduces asymmetric aimethlarginine (ADMA) levels, and increases endothelial NO synthase (eNOS) phosphorylation and NO production in cultured HUVECs, vascular endothelium of atherosclerotic lesions and liver. In contrast, systemic delivery of miR‐21 in *Apoe^−^* ^/^ *^−^* mice or miR‐21 overexpression in cultured HUVECs abrogates those DMY‐mediated protective effects. These data demonstrate that endothelial miR‐21‐inhibited DDAH1‐ADMA‐eNOS‐NO pathway promotes the pathogenesis of atherosclerosis which can be rescued by DMY. Thus, DMY may represent a potential therapeutic adjuvant in atherosclerosis management.

## 1 INTRODUCTION

Endothelial cell (EC) activation and dysfunction is the initial step that plays a critical role in the pathogenesis of atherosclerosis.^1^, ^2^, ^3^ For example, activated ECs express multiple adhesion molecules, such as vascular cell adhesion molecule‐1 (VCAM‐1) and intercellular adhesion molecule‐1 (ICAM‐1), to facilitate the circulating leukocytes into the vascular wall, where they differentiate into macrophages and eventually become foam cells.^1^, ^4^, ^5^ Accumulating studies demonstrate that reduced nitric oxide (NO) production, caused by impaired endothelial NO synthase (eNOS) function, is a hallmark of EC dysfunction that contributes importantly to atherosclerosis.^3^, ^6^ Asymmetrical dimethylarginine (ADMA), mainly metabolized by dimethylarginine dimethylaminohydrolase‐1 (DDAH1), is the foremost endogenous NOS inhibitor through directly competing with the physiological precursor L‐arginine from the enzyme.^7^, ^8^ Recently, the DDAH1‐ADMA‐eNOS pathway has emerged as an important regulator in mediating NO production and atherosclerosis. The elevated levels of ADMA are positive correlated with all established cardiovascular risk factors and acted as a biomarker and/or inducer of endothelial dysfunction.^9^ Overexpression of DDAH1 increases NO production and improves endothelial function through an ADMA‐dependent manner.^10^ Moreover, genetic overexpression of DDAH1 reduces atherosclerotic plaque formation in apolipoprotein E‐deficient (*Apoe^−^* ^/^ *^−^*) mice by lowering ADMA levels and improving endothelial function.^11^ Thus, targeting DDAH1‐ADMA–mediated eNOS‐NO activation and associated endothelial function holds great potential for developing novel therapeutic approaches for atherosclerosis.

Accumulating evidence demonstrates that traditional Chinese herbal medicines are emerging as promising therapeutic drugs for treating cardiovascular diseases. For instance, *Ampelopsis grossedentata*, also known as vine tea and widely distributed in southern China, has been consumed as health tea and herbal medicine for hundreds of years.^12^ Dihydromyricetin (DMY) is the most abundant and bioactive flavanonol compound that can be robust extracted from the stems and leaves of *A grossedentata* using response surface methodology.^13^ Recent studies demonstrated that DMY attenuates both pressure overload‐ and angiotensin II‐induced cardiac hypertrophy through ameliorating oxidative stress reaction.^14^, ^15^ In addition, in a mouse model of acute myocardial infarction, DMY reducesischemia/reperfusion‐induced cardiomyocytes apoptosis, resulting in less infarct area and the improvement of cardiac function.^16^ Moreover, DMY increases glucose uptake in skeletal muscle, thereby improving insulin resistance, a major risk factor in the development of cardiovascular diseases.^17^ Though it has been reported that DMY ameliorates atherosclerosis,^18^, ^19^ the signals and molecular mechanisms of how DMY attenuates endothelial function, vascular inflammation and atherosclerosis are largely unknown.

MicroRNAs (miRNAs), such as miR‐21, have emerged as important regulators of endothelial activation and dysfunction that contribute importantly to the development of atherosclerosis.^20^, ^21^, ^22^ Our previous studies identified that miR‐21 was increased in ECs in response to tumour necrosis factor alpha (TNF‐α) and 4‐hydroxynonenal (4‐HNE) stimulation and miR‐21–mediated DDAH1‐ADMA‐eNOS activation plays a critical role in mediating DMY’s protective effects on TNF‐α–induced endothelial dysfunction.^20^, ^23^ In this study, we found that DMY decreases miR‐21 expression, improves EC function and thereby inhibits vascular inflammation, lipid metabolism and atherosclerosis in *Apoe^−^* ^/^ *^−^* mice. We identified an important role of endothelial miR‐21‐DDAH1‐ADMA‐eNOS‐NO signalling in DMY‐ameliorated atherosclerotic lesion formation, indicating that DMY supplementation may serve as a potential therapeutic adjuvant for treating atherosclerosis.

## 2 MATERIALS AND METHODS

### 2.1 Animal studies

All animal procedures were approved by the Institutional Animal Care and Use Committee at Second Xiangya Hospital, Central South University. Male *Apoe^−^* ^/^ *^−^* mice and C57BL/6J mice from 8‐ to 10‐week‐old were purchased from the Beijing Vital River Laboratory Animal Technology Co. in China. All mice were maintained on a 12‐hour light/dark cycle in a pathogen‐free animal facility. Mice were kept on a standard chow diet or on a 1.25% high cholesterol diet (HCD; D12108C, Research Diets) for 12 weeks. Mice had free access to food and water. For DMY intervention study, *Apoe^−^* ^/^ *^−^* mice were administered daily an intragastric gavage with DMY (500 mg/kg; D101549, Aladdin; n = 8), DMY puls L‐NAME (50 mg/kg; N5751, Sigma; n = 7) or same dosage of solution control (n = 10). For in vivo systemic overexpression of miR‐21 efficient assay experiment, C57BL/6N mice were treated with miR‐21 mimics (21‐m; miR10000076‐1‐5, RiboBio) or miRNA non‐specific control (NS‐m; miR1N0000001‐1‐5, RiboBio) for two consecutive days (once a day, 20 nmol/injection, iv) and harvested after 7 days (n = 3 for each group). For in vivo miR‐21 accumulation assay, 8‐week‐old male *Apoe^−^* ^/^ *^−^* mice were kept on a HCD for 4 weeks followed by tail vein injection of FITC‐labelled or unlabelled miR‐21 mimic (20 nmol/injection, iv) and harvested 4 hours after injection. For miR‐21 intervention study, *Apoe^−^* ^/^ *^−^* mice were kept on a HCD and daily intragastric gavage with DMY (500 mg/kg) for 12 weeks. Eight weeks after HCD, mice were tail vein injected with 21‐m or NS‐m for two consecutive days and then followed by once a week for 3 weeks (20 nmol/injection; n = 7 for each group). Systemic delivery of miRNA was performed according to the established protocol described in Ref. 24. Briefly, 20 nmol 21‐m or NS‐m was dissolved in 100 μL dPBS (solution 1). Lipofectamine 2000 (30 μL; 11668019, Invitrogen) was mixed with 70 μL dPBS by pipetting up and down (solution 2), and placed at room temperature for 15 minutes. Then, solution 1 and solution 2 were mixed by pipetting up and down. After incubating at room temperature for 30 minutes, the mixture (200 μL) was injected into mice by tail vein injection. All mice in the current study were randomly assigned to groups. After 12 weeks, mice were humanely killed, followed by cardiac puncture blood collection, and aortic root and liver were harvested. Aortic roots were embedded in optimum cutting temperature (OCT) compound and frozen at −80°C, while part of liver was fixed in 4% paraformaldehyde (PFA) and the rest were frozen at −80°C for further experiments.

### 2.2 Atherosclerotic lesions characterization and immunohistological analysis

Serial cryostat sections (6 μm) from OCT‐embedded aortic roots were prepared using a Lab‐Tek tissue processor Leica CM1950. Paraffin sections (6 μm) were prepared from 4% PFA fixed liver tissues. To determine atherosclerotic lesion size, aortic root sections and the descending thoracoabdominal aorta were stained with oil red O.

For immunohistological analysis, serial cryostat sections from aortic root were fixed and permeabilized with cold‐acetone for 5 minutes and blocked in PBS containing 10% normal goat serum for 1 hour at room temperature. Paraffin sections from liver were deparaffinized, and antigen retrieval was performed using citrate buffer purchased from Abcam (ab93678). Then, sections were stained for macrophages (anti‐Mac‐2, 1:100, CL8942AP, Cedarlane), T cells (anti‐CD4, 1:100, 553043, BD Pharmingen) antibodies for 1.5 hours, followed by appropriated biotin‐conjugated secondary antibodies (1:1000, SA00004‐8, Proteintech) and HRP‐streptavidin (CW2069S, CWBIO). CD4‐positive T cells were counted and presented as numbers of total cells per lesion. Mac‐2–positive areas were measured using computer‐assisted Image‐Pro Plus software (Meida Cybernetics) and presented as positive area per μm^2^ of liver area.

For immunofluorescence staining, aortic root sections were stained with rat anti‐Mac‐2 (1:100, CL8942AP; Cedarlane), FITC‐labelled anti‐actin, α‐smooth muscle (α‐SMA; 1:500, F3777; Sigma), rat anti‐CD31 (1:200, ab23864; Abcam), rabbit anti‐VCAM1 (1:250, ab134047; Abcam), rabbit anti‐p‐eNOS (ser1177; 1:250, ab184154; Abcam) and rabbit anti‐DDAH1 (1:250, ab180599; Abcam) for 3 hours at room temperature, followed by incubation with Alexa Fluor 555 (1:300, A21434; Invitrogen) or 488 (1:300, A11034; Invitrogen) labelled secondary antibody for 1 hour at room temperature. DPAI (P36935; Invitrogen) was used to stain the nuclei. All images were captured by Nikon A1 confocal laser scanning microscopy (Nikon, Japan). Mac‐2–positive and SMA‐positive area were measured using Image‐Pro Plus software and present as positive area per μm^2^ of lesion area. P‐eNOS, DDAH1 and VCAM‐1 expression in CD31‐positive ECs were measured using Image‐Pro Plus software and present as mean intensity per length of endothelium in aortic root.

### 2.3 Intima RNA isolation from aorta tissue

Intima RNA isolation from aorta was performed according to established protocol described in a previous study.^24^ Briefly, aortas were gently flushed with ice‐cold PBS, followed by intima peeling using TRIzol reagent (15596018, Invitrogen). TRIzol was carefully flushed for 10, and 10 seconds pause, followed by another 10 seconds flushed and collected in an RNA‐free Eppendorf tube and snap‐frozen in liquid nitrogen.

### 2.4 Plasma RNA isolation

Total RNA isolated from plasma was isolated using total RNA purification kit (37500, Norgen Biotek) according to the manufacturer's instruction. During the RNA isolation procedure, miR‐39‐3p (miRB0000010‐3‐1) was added as an endogenic control.

### 2.5 Lipid profile analysis

The plasma levels of total cholesterol (A111‐1‐1; Nanjing Jiancheng Bioengineering), triglyceride (A110‐1‐1; Nanjing Jiancheng Bioengineering), low‐density lipoprotein cholesterol (LDL‐c; A113‐1‐1; Nanjing Jiancheng Bioengineering) and high‐density lipoprotein cholesterol (HDL‐c; A112‐1‐1; Nanjing Jiancheng Bioengineering) were measured using commercial colorimetric enzymatic assay kits according to the manufacturers’ recommendation.

### 2.6 Plasma and liver ADMA and NO levels measurement

Same volume of plasma samples were used for measuring AMDA (E‐EL‐0042c; Elabscience) and NO (S0023; Beyotime) levels from mice using the relevant commercial ELISA kits according to manufacturers’ instruction. To detect AMDA and NO concentrations in liver, equal amount of protein from each mouse were measured by using the relevant AMDA and NO kit.

### 2.7 Cell culture

Human umbilical vein ECs (HUVECs) were obtained from (8000; ScienCell) and cultured in EC growth medium EGM‐2 (cc‐3162; Lonza). THP‐1 cells were purchased from ATCC (TIB‐202) and cultured in RPMI 1640 medium (SH30027FS; Hyclone) supplemented with 10% foetal bovine serum (16140071; Gibco) and 0.05 mmol/L 2‐mercaptoethanol (M6250; Sigma). Cells passaged less than six times were used for all experiments. HUVECs were plated 10 000/well on a 96‐well fluorescence plate (353948, BD) for NO probe detection and cell adhesion assay, 70 000/well on a 12‐well plate for RNA isolation, ADMA levels and intracellular and extracellular NO measuring studies, and 150 000/well on a six‐well plate for protein isolation, respectively. Cells were grown to 80%‐90% confluency and then pretreated with DMY for 10 hours. Afterwards, HUVECs were stimulated with 120 μg/mL of human ox‐LDL (UnionBiol), or ox‐LDL plus DMY (25 μmol/L), or ox‐LDL plus DMY and NOS inhibitor L‐NAME (25 μmol/L) for 16 hours and then harvested for indicated experiments. For miR‐21 gain‐of‐function experiments, miR‐21 agomir (miR40000076‐4‐5; RiboBio) and agomir NC (miR4N0000001‐4‐5; RiboBio) were transfected at 100 nm as previous described in Ref. [20]. Then transfected cells were treated with ox‐LDL, or ox‐LDL plus DMY as described above.

### 2.8 Cell adhesion assay

Cell adhesion assay was performed as previously described in Ref. [25]. Briefly, HUVECs were plated on a 96‐well fluorescence plate and treated as described in the cell culture methods part. THP‐1 cells were washed with serum‐free RPMI 1640 medium and suspended at 5 × 10^6^ cell/mL in serum‐free medium with 5 µmol/L of Calcein AM (C3100MP; Invitrogen). Cells were incubated for 30 minutes and the labelling reaction was stopped by adding the same volume of cell growth medium. Then cells were washed with growth medium twice and resuspended in growth medium at 5 × 10^5^ cell/mL. After 16 hours of the relevant treatment, HUVECs were washed once with THP‐1 cell growth medium, and 200 µL Calcein AM‐labelled THP‐1 cells were added to each well. After 1 hour of incubation, the non‐adherent cells were removed carefully and adherent cells were washed with RPIM 1640 medium five times. The images were captured by a microscopy (Carl Zeiss). The mean fluorescence intensity per view was used to show the numbers of THP‐1 cells attached to ECs and quantified from randomly acquired images.

### 2.9 Measurement of intracellular NO production

The production of NO in HUVECs was examined using commercial DAF‐FM DA kit (S0019; Beyotime) according to the manufacturers’ instruction. Briefly, HUVECs after treatment were washed three times in PBS and then incubated with DAF‐FM DA (5 µmol/L) in PBS for 20 minutes at 37°C. After incubation, cells were washed three times in PBS and images were captured by a microscopy (Carl Zeiss). The mean fluorescence intensity per view was used to show the NO production in ECs and quantified from randomly acquired images.

### 2.10 Real‐time qPCR

Tissues were homogenized using TissueLyser II (QIAGEN) according to the manufacture's instruction. Total RNA was isolated using TRIzol reagent (15596018; Invitrogen) from homogenized liver and cultured HUVECs. PrimeScript RT reagent kit (RR047A; Takara) was used to generate cDNA, and TB Green Premix EX Tag kit (RR820A; Takara) was used for real‐time qPCR with the Real‐time PCR system (Roche) following the manufacturer's instruction. Specific primers including miR‐21‐5p (MQPS0000835‐1‐200), U6 (MQPS0000002‐1‐100) and miR‐39‐3p (miRA0000010‐1‐100) were purchased from RiboBio. Primers are listed in Table S1.

### 2.11 Protein extraction and immunoblot

Tissues were homogenized using TissueLyser II (QIAGEN) according to the manufacture's instruction. Homogenized liver tissue and cultured HUVECs were isolated RIPA buffer supplemented with protease inhibitor (CW2200, CWBIO) and phosphorylase inhibitor (CW2383, CWBIO). Lysates were separated by 10% SDS‐PAGE gels, transferred to PVDF membranes (IPVH00010; Millipore) and blocked in 5% non‐fat milk in TBST for 1 hour at room temperature. Then, membranes were incubated with the relevant antibodies including rabbit anti‐DDAH1 (1:1000, ab180599; Abcam), rabbit anti‐phospho‐eNOS (1:1000, ab184154; Abcam), rabbit anti‐GAPDH (1:1000, 2118s; CST) and rabbit anti‐VCAM‐1 (1:1000, ab134047; Abcam) overnight at 4°C. Proteins were visualized by ECL Plus Western blotting detection reagents (RPN2132; GE).

### 2.12 Statistical analysis

GraphPad 7.0 software package (GraphPad Software, Inc) was used for statistical analysis. Unpaired two‐tailed Student's *t* test was used to determine statistical significance between two groups for normally distributed continuous variables. For multiple groups comparison, one‐way analysis of variance (ANOVA) followed by Tukey multiple comparison analysis were used. For data without normal distribution, non‐parametric Mann‐Whitney *U* test or Kruskal‐Wallis test was used. All data were expressed as mean ± SEM *P* < .05 was considered significant for all tests.

## 3 RESULTS

### 3.1 DMY inhibits atherosclerosis by increasing NO production and improving endothelial function in *Apoe^−^* ^/^ *^−^* mice

**FIGURE 1:**
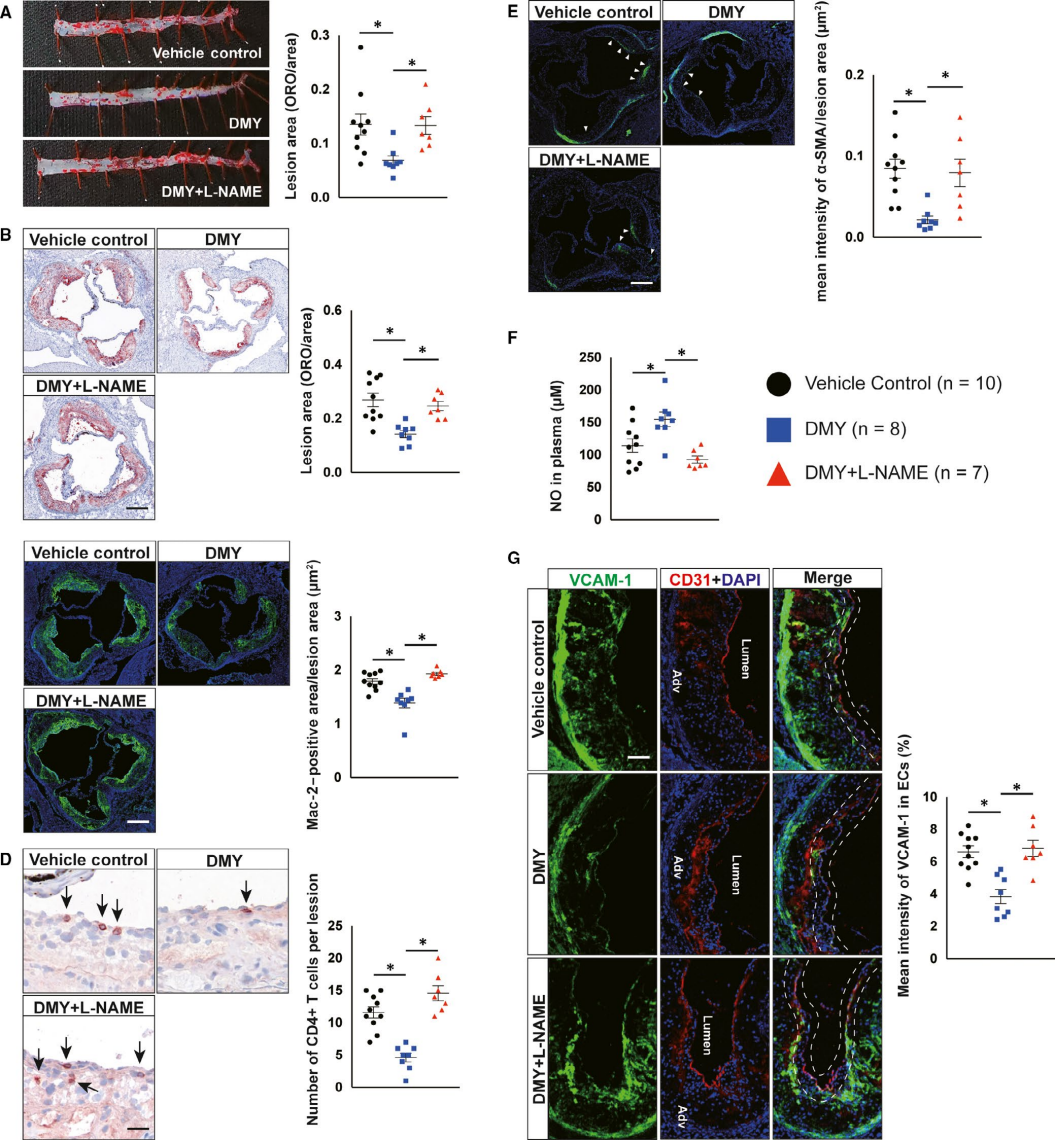
Inhibition of nitric oxide (NO) production by L‐NAME abrogates the inhibitory effects of dihydromyricetin (DMY) on atherosclerosis in *Apoe^−^* ^/^ *^−^* mice. A, Lesion areas were quantified by Oil Red O (ORO)‐stained thoracoabdominal aorta. B, Lesion areas were quantified by ORO‐stained aortic sinus sections. Scale: 200 μm. C, D, Representative images and quantification show Mac‐2–positive macrophages (C) and CD4‐positive T cells (D) in the aortic sinus lesions. Scale: 200 μm (C) and 100 μm (D). Arrows indicate CD4‐positve T cells in aortic sinus lesions. E, Representative images and quantification show α‐smooth muscle actin (α‐SMA)‐positive smooth muscle cells (SMCs) accumulation in the aortic sinus lesions. Arrows indicate differential SMCs accumulation in aortic sinus lesions. Scale: 200 μm. F, ELISA analysis of NO levels in plasma from vehicle, DMY or DMY combined with L‐NAME–treated *Apoe^−^* ^/^ *^−^* mice. G, Representative images and quantification show VCAM‐1 expression in endothelial cells in the aortic sinus lesions. Frozen sections of aortic sinus were stained for anti‐VCAM‐1 (green), anti‐CD31 (red) and 4′,6‐diamidino‐2‐phenylindole (DAPI; blue). The dashed line area indicates differential VCAM‐1 expression in endothelial cells. Scale: 100 μm. Data shown are mean ± SEM (n = 7‐10 mice per group). *P < .05

Nitric oxide‐dependent endothelial dysfunction contributes importantly to the pathogenesis of atherosclerosis.^3^, ^6^ We previously found that DMY‐ameliorated TNF‐α–induced dysfunction of HUVECs by increasing NO generation.^20^ To investigate whether DMY attenuates atherosclerosis through NO production, *Apoe^−^* ^/^ *^−^* mice were fed a HCD for 12 weeks and administered daily intragastric gavage with vehicle, DMY or DMY plus NOS inhibitor L‐NAME, respectively. As shown in Figure 1A,B, analysis of atherosclerotic lesion formation by Oil Red O staining revealed a 49% reduction in lesion area in the descending thoracoabdominal aorta and a 45% reduction in lesion size at the aortic sinus in DMY‐treated *Apoe^−^* ^/^ *^−^* mice compared to *Apoe^−^* ^/^ *^−^* mice treated with vehicle. Histological assessment of atherosclerotic lesions at the aortic sinus revealed a 22% reduction in macrophages content by Mac‐2 staining (Figure 1C), a 60% reduction in T cells content by CD4 staining (Figure 1D) and a 75% reduction in smooth muscle cells (SMCs) accumulation (Figure 1E), respectively, in DMY‐treated *Apoe^−^* ^/^ *^−^* mice compared with vehicle‐treated *Apoe^−^* ^/^ *^−^* mice. The DMY‐mediated inhibition of atherosclerotic lesion formation and inflammatory content were associated with an increase in NO levels (Figure 1F) and an inhibition of endothelial activation indicated by reduced VCAM‐1 expression in CD31‐positive ECs at aortic sinus (Figure 1G). Consistent with these observations, NOS inhibitor L‐NAME treatment lowered NO concentrations and completely blocked the beneficial effects of DMY on atherosclerotic lesion formation in *Apoe^−^* ^/^ *^−^* mice and endothelial function (Figure 1), suggesting DMY improves endothelial function and attenuates atherosclerosis in a NO‐dependent manner. In support of this, pretreatment with DMY significantly increased NO generation and secretion (Figure S1A‐C), reduced VCAM‐1 protein and mRNA and ICAM‐1 and E‐selectin mRNA expression in HUVECs (Figure S1D,E) and the numbers of monocytes attached to HUVECs (Figure S1F) in response to ox‐LDL stimulation. In contrast, L‐NAME abrogated DMY’s protective effects on ox‐LDL–induced EC dysfunction (Figure S1). Taken together, these results suggest that DMY inhibits endothelial activation and reduces macrophages and CD4–positive T cells accumulation in the vascular wall, resulting in the inhibition of atherosclerotic lesion formation in *Apoe^−^* ^/^ *^−^* mice, at least in part, by increasing NO generation in ECs.

### 3.2 DMY improves lipid metabolism and hepatic inflammation by increasing NO production in *Apoe^−^* ^/^ *^−^* mice

**FIGURE 2:**
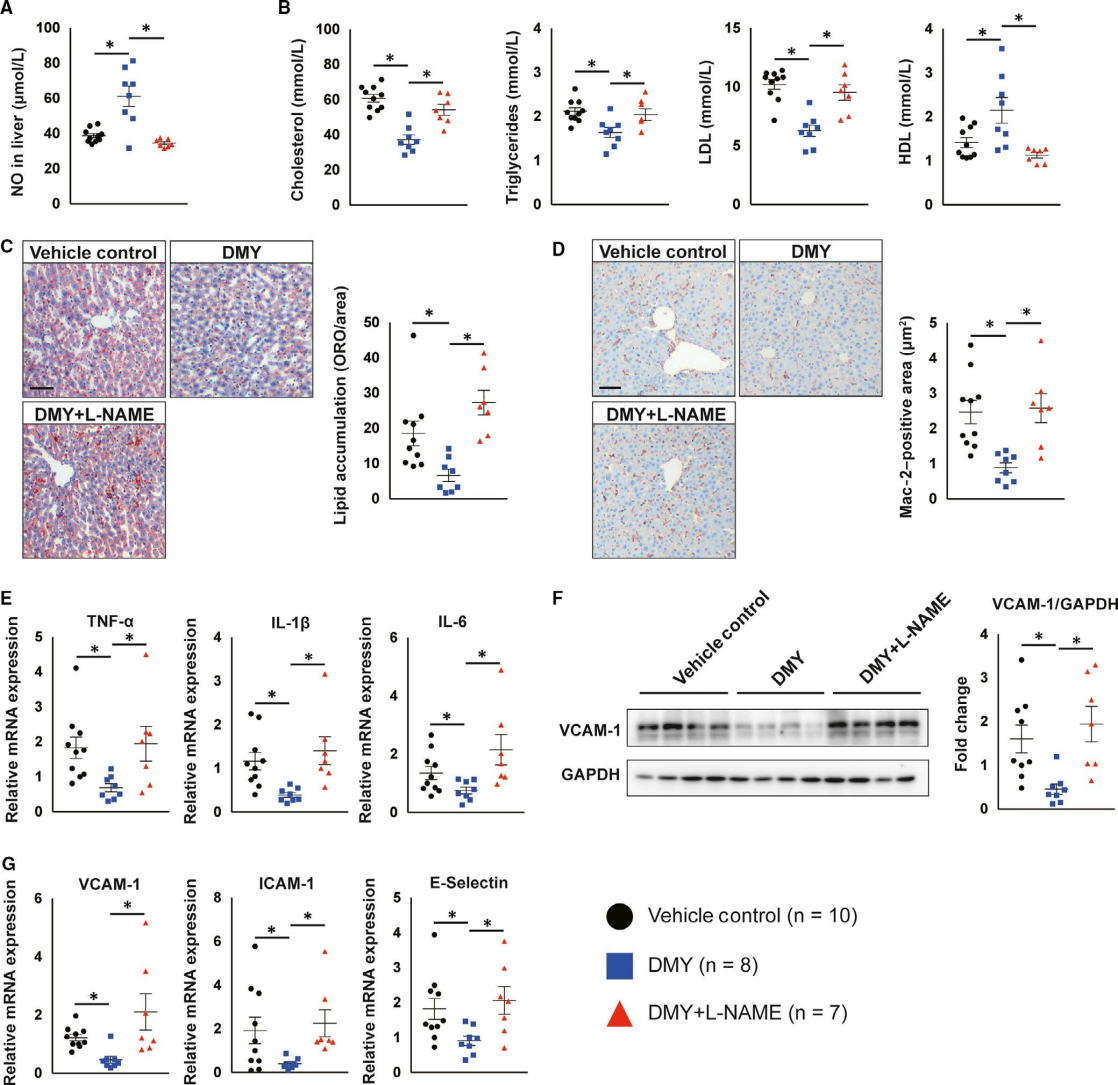
Dihydromyricetin (DMY) attenuates plasma lipid levels and hepatic endothelial activation and inflammation depending on nitric oxide (NO) production in *Apoe^−^* ^/^ *^−^* mice. A, ELISA analysis of NO levels in livers from vehicle control, DMY or DMY combined with L‐NAME–treated *Apoe^−^* ^/^ *^−^* mice fed with HFD for 12 wk. B, Circulating lipid levels (total cholesterol, triglycerides, LDL‐C, HDL) in HFD‐fed *Apoe^−^* ^/^ *^−^* mice treated with vehicle control, DMY or DMY combined with L‐NAME after 12 wk. C, Representative images and quantification show lipid accumulation in livers from vehicle control, DMY or DMY combined with L‐NAME–treated *Apoe^−^* ^/^ *^−^* mice fed with HFD for 12 wk. Scale: 100 μm. D, Representative images and quantification show Mac‐2–positive macrophages in livers. Scale: 100 μm. E, Real‐time qPCR analysis of indicated macrophage M1 markers in liver. The expression of genes was normalized to mouse β‐actin. F, Western blot analysis of VCAM‐1 expression in liver. G, Real‐time qPCR analysis of indicated endothelial cells activated markers in liver. The expression of genes was normalized to mouse β‐actin. Data shown are mean ± SEM (n = 7‐10 mice per group). *P < .05

Atherosclerosis is a complex process involving multiple steps and the interplay of systemic and local factors. Clinical and experimental evidence demonstrated that plasma cholesterol, particularly low‐density LDL, is a critical driver in atherosclerotic plaque initiation and progression.^26^, ^27^ Compared with vehicle‐treated *Apoe^−^* ^/^ *^−^* mice, the NO levels in liver were higher in DMY‐treated *Apoe^−^* ^/^ *^−^* mice by 1.35‐fold (Figure 2A). Moreover, we observed that *Apoe^−^* ^/^ *^−^* mice received DMY treatment had lower plasma cholesterol, triglyceride and LDL levels by 39%, 23% and 39%, respectively, while had higher levels of HDL by 151% compared with *Apoe^−^* ^/^ *^−^* mice receiving vehicle control (Figure 2B). Furthermore, Oil Red O staining in livers from vehicle control and DMY‐treated *Apoe^−^* ^/^ *^−^* mice revealed a 64% reduction in lipid accumulation in *Apoe^−^* ^/^ *^−^* mice treated with DMY (Figure 2C). L‐NAME treatment significantly decreased NO levels and abrogated DMY‐improved lipid metabolism and accumulation in *Apoe^−^* ^/^ *^−^* mice treated with DMY (Figure 2A‐C). Hepatic macrophages play a critical role in maintaining liver homeostasis and the pathogenesis of hepatic steatosis, leading to abnormal lipid metabolism and elevation of circulating lipid levels.^28^ This prompted us to examine whether these protective effects of DMY on lipid metabolism are the consequence of attenuated hepatic inflammation and whether those phenotypic changes depend on improved endothelial NO production and function in DMY‐treated *Apoe^−^* ^/^ *^−^* mice. We observed that macrophage content indicated by Mac‐2 staining was significantly reduced by 64% in the livers from DMY‐treated *Apoe^−^* ^/^ *^−^* mice compared with vehicle‐treated *Apoe^−^* ^/^ *^−^* mice (Figure 2D). Macrophage M1 markers, such as TNF‐α, IL‐1β and IL‐6, were also reduced in the livers from those DMY‐treated *Apoe^−^* ^/^ *^−^* mice (Figure 2E). In contrast, the macrophage content and macrophage M1 markers were not alerted in *Apoe^−^* ^/^ *^−^* mice treated with DMY plus L‐NAME (Figure 2D,). Furthermore, we observed that DMY treatment reduced VCAM‐1 protein and VCAM‐1, ICAM‐1 and E‐Selectin mRNA expression in the livers from HCD‐fed *Apoe^−^* ^/^ *^−^* mice (Figure 2F,G), suggesting that EC dysfunction in liver was ameliorated by DMY administration. These differences were abolished by L‐NAME treatment (Figure 2D‐G). Taken together, these data indicate that DMY treatment improves endothelial function, hepatic inflammation and lipid metabolism by increasing NO production.

### 3.3 DMY increases endothelial NO generation through increasing DDAH1‐ADMA‐eNOS pathway activation

**FIGURE 3:**
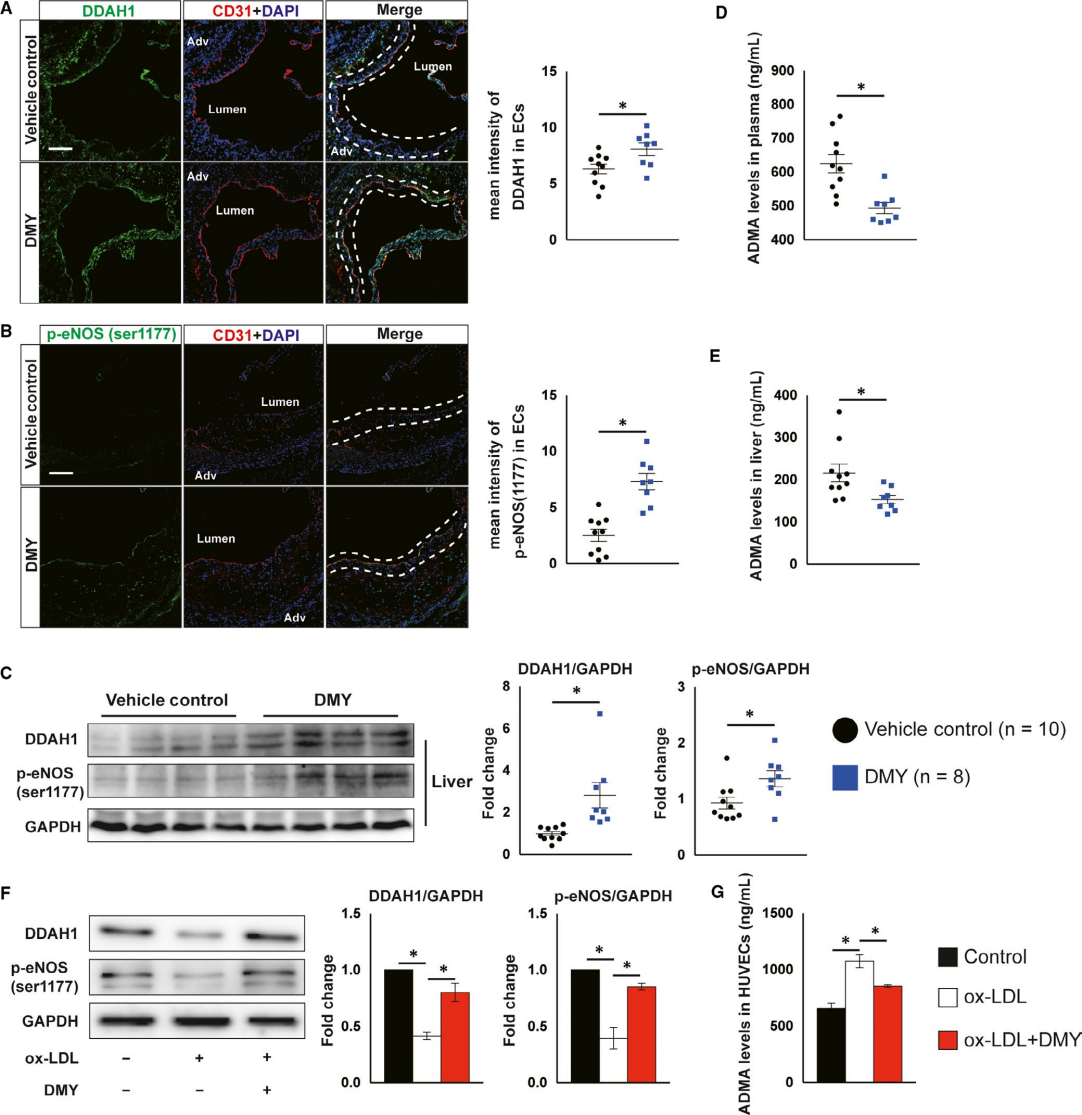
Dihydromyricetin (DMY) administration increases DDAH1/endothelial nitric oxide synthase (eNOS) activation in vivo and in vitro. A, B, Representative images and quantification show DDAH1 (A) and phosphor‐eNOS (ser1177) (B) expression in endothelial cells in the aortic sinus lesions rom vehicle control, DMY‐treated *Apoe^−^* ^/^ *^−^* mice fed with HFD for 12 wk. Frozen sections of aortic sinus were stained for anti‐DDAH1 or phosphor‐eNOS (green), anti‐CD31 (red) and 4′,6‐diamidino‐2‐phenylindole (DAPI; blue). The dashed line area indicates differential DDAH1 or phosphor‐eNOS expression in endothelial cells. Scale: 50 μm. n = 8‐10 mice per group. C, Western blot analysis of DDAH1 and p‐eNOS (ser1177) expression in liver. n = 8‐10 mice per group. D, E, ELISA analysis of ADMA levels in plasma (D) and livers (E) from vehicle control, DMY‐treated *Apoe^−^* ^/^ *^−^* mice fed with HFD for 12 wk. n = 8‐10 mice per group. HUVECs were treated with ox‐LDL (120 μg/mL) or ox‐LDL plus DMY (25 μmol/L) for 16 hours and harvested for indicated experiments. F, Western blot analysis of DDAH1 and p‐eNOS (ser1177) expression. n = 3 independent experiments. G, ELISA analysis of ADMA levels in cell lysates. n = 3 independent experiments. Data shown are mean ± SEM. *P < .05

In ECs, NO is synthesized directly by eNOS through metabolizing substrate L‐arginine. During this process, DDAH1 plays an important role in controlling eNOS activation by catalyzing the endogenous eNOS inhibitor ADMA.^29^ Thus, we next investigated whether DMY promotes NO production by regulating the DDAH1‐ADMA‐eNOS pathway. In atherosclerotic lesions from aortic sinus, immunofluorescent double staining assay revealed that the expression of DDAH1 and phosphorylation of eNOS (ser1177) in ECs were significantly increased in DMY‐treated *Apoe^−^* ^/^ *^−^* mice in comparison with mice treated with vehicle (Figure 3A,). Moreover, immunoblot assay showed that DMY treatment increased DDAH1 and phospho‐eNOS (ser1177) expression by 285% and 147%, respectively, in liver from DMY‐treated *Apoe^−^* ^/^ *^−^* mice compared with vehicle‐treated *Apoe^−^* ^/^ *^−^* mice (Figure 3C). Furthermore, *Apoe^−^* ^/^ *^−^* mice treated with DMY decreased the levels of ADMA in both plasma and liver tissue (Figure 3D,). Finally, in HUVECs exposed to ox‐LDL, DMY pretreatment increased DDAH1 protein expression and phosphorylation of eNOS (ser1177) and decreased the intracellular ADMA levels, respectively, compared with control cells (Figure 3F,G). Collectively, these data suggest that DMY increases endothelial NO generation and improves endothelial function by activating DDAH1‐ADMA‐eNOS pathway.

### 3.4 Systemic delivery of miR‐21 decreases NO generation and blocks DMY’s protective effects on atherosclerotic plaque formation, lipid metabolism and hepatic inflammation in HCD‐fed *Apoe^−^* ^/^ *^−^* mice

MiR‐21 is a conserved small non‐coding RNA in human and mice, and plays a critical role in the pathogenesis of atherosclerosis. We have shown that DMY improves TNF‐α–induced endothelial dysfunction by decreasing miR‐21 expression.^20^ Moreover, compared with vehicle‐ treated *Apoe^−^* ^/^ *^−^* mice, the miR‐21 expression was decreased by 47% in plasma and 41% in liver, respectively, in those DMY‐treated *Apoe^−^* ^/^ *^−^* mice (Figure S2A,B). Furthermore, in HUVECs in response to ox‐LDL, miR‐21 expression was significantly increased compared with control, as measured by qPCR, which was attenuated by DMY (Figure S2C). To examine whether DMY inhibits atherosclerosis by down‐regulating miR‐21 expression, we first tested whether systemic delivery of exogenous miR‐21 mimics (21‐m) by tail vein injection could increase miR‐21 expression in EC‐enriched aortic intima and liver. As shown in Figure S3A,B, the expression of miR‐21 in the endothelial enriched aortic intima and liver from C57BL/6J mice received two consecutives daily tail vein injection of miR‐21 (20 nmol/injection) were 2.1‐fold and 7.6‐fold higher than that of mice injected with the miRNA non‐specific control (NS‐m), respectively, on day 7 after injection. These data indicate that exogenous miR‐21 administered by tail vein injection is able to accumulate in the aortic intima and liver. Moreover, using FITC‐labelled 21‐m, we found that miR‐21 accumulated in ECs and sub‐endothelial area in atherosclerotic plaque in aortic sinus from *Apoe^−^* ^/^ *^−^* mice fed with HCD after 4 weeks (Figure S3C,D).

**FIGURE 4:**
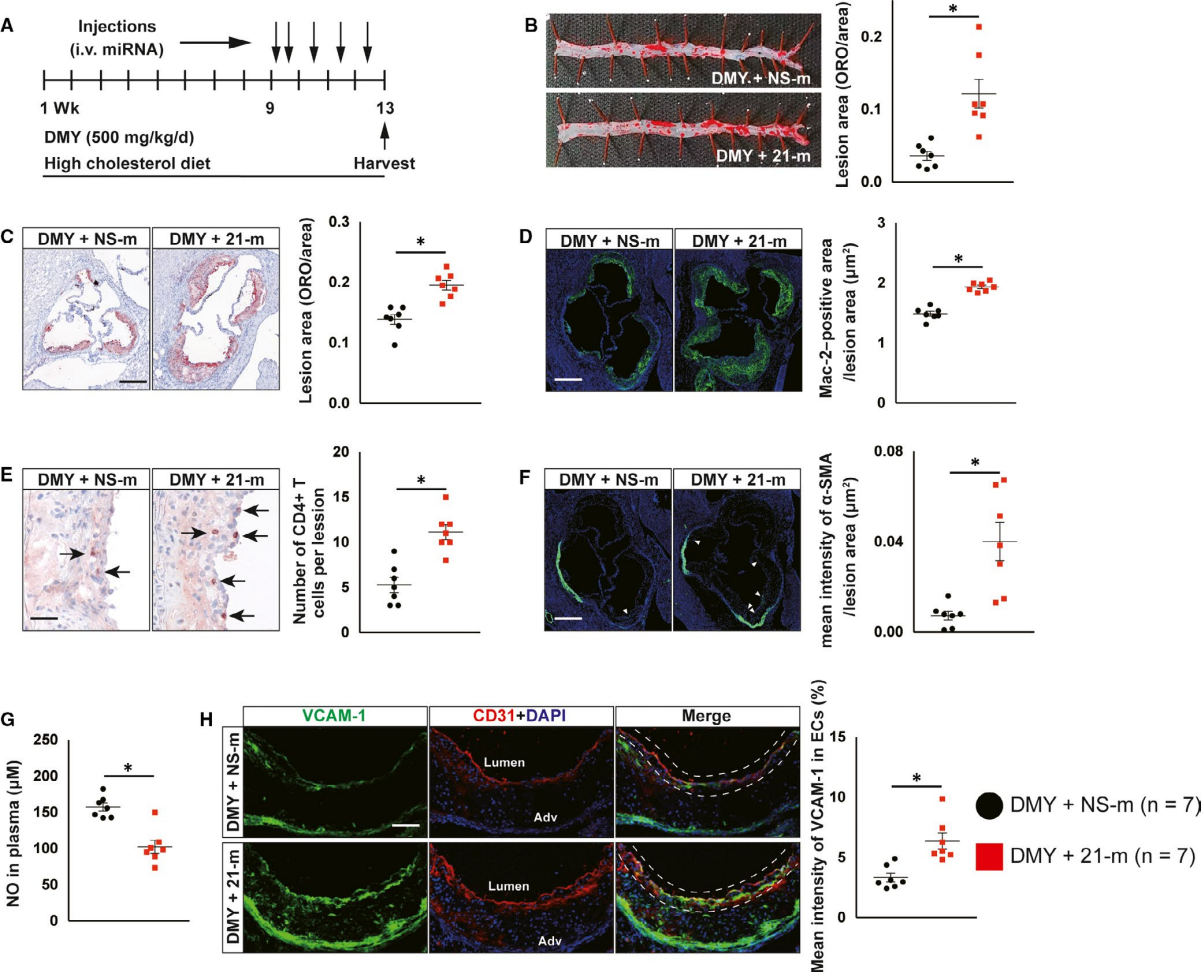
Systemic delivery of miR‐21 mimics blocks the protective effect of dihydromyricetin (DMY) on atherosclerosis in *Apoe^−^* ^/^ *^−^* mice. A, Schema of experimental procedure. B, Lesion areas were quantified by Oil Red O (ORO)‐stained thoracoabdominal aorta. C, Lesion areas were quantified by ORO‐stained aortic sinus sections. Scale: 200 μm. D, E, Representative images and quantification show Mac‐2–positive macrophages (D) and CD4‐positive T cells (E) in the aortic sinus lesions. Scale: 200 μm (D) and 100 μm (E). Arrows indicate CD4‐positve T cells in aortic sinus lesions. F, Representative images and quantification show α‐smooth muscle actin (α‐SMA)‐positive smooth muscle cells (SMCs) accumulation in the aortic sinus lesions. Arrows indicate differential SMCs accumulation in aortic sinus lesions. Scale: 200 μm. G, ELISA analysis of nitric oxide levels in plasma from DMY combined with NS‐m injection or DMY combined with 21‐m injection‐treated *Apoe^−^* ^/^ *^−^* mice. H, Representative images and quantification show VCAM‐1 expression in endothelial cells in the aortic sinus lesions. Frozen sections of aortic sinus were stained for anti‐VCAM‐1 (green), anti‐CD31 (red) and 4′,6‐diamidino‐2‐phenylindole (DAPI; blue). The dashed line area indicates differential VCAM‐1 expression in endothelial cells. Scale: 100 μm. Data shown are mean ± SEM (n = 7 mice per group). *P < .05

**FIGURE 5:**
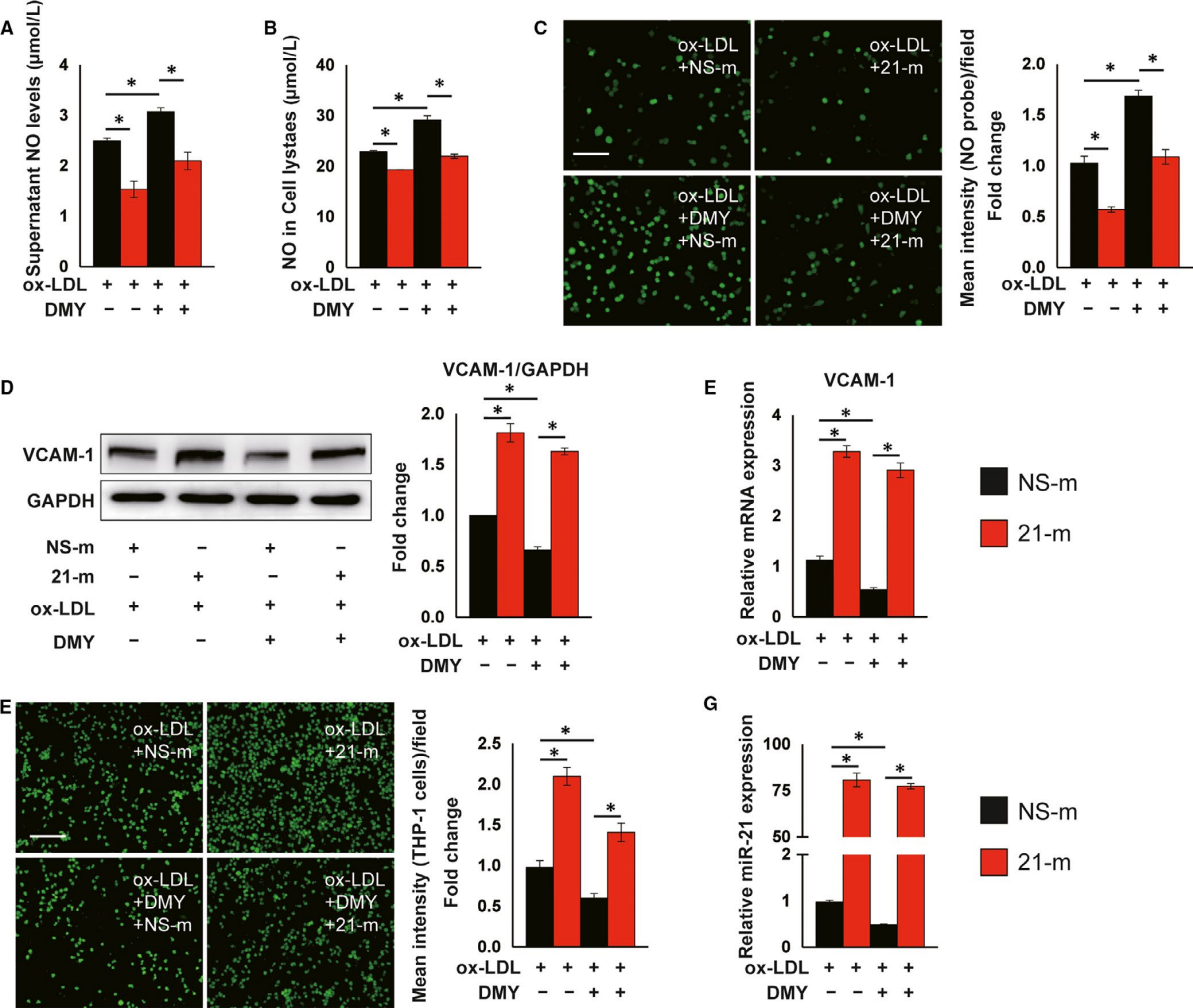
Overexpression of miR‐21 inhibits nitric oxide (NO) production and abrogates dihydromyricetin (DMY)‐attenuated EC activation in response to ox‐LDL. HUVECs were transfected with 100 nm agomir NC or miR‐21 agomir for 24 h and then treated with ox‐LDL (120 μg/mL) or ox‐LDL plus DMY (25 μmol/L) for 16 h and harvested for indicated experiments. A and B, ELISA analysis of NO levels in supernatant (A) and cell lysates (B). n = 3 independent experiments. C, Fluorescent NO probe analysis of NO levels in live HUVECs. Scale: 50 μm. n = 3 independent experiments. D, Western blot analysis of VCAM‐1 expression. n = 3 independent experiments. E, Real‐time qPCR analysis of VCAM‐1. Expression of VCAM‐1 was normalized to GAPDH. n = 3 independent experiments. F, Representative images and quantification show THP‐1 cells adhering to HUVECs after treatment. Scale: 100 μm. n = 3 independent experiments. G, Real‐time qPCR analysis of miR‐21. Expression of miR‐21 was normalized to U6. n = 3 independent experiments. Data shown are mean ± SEM. *P < .05

Thus, we next performed another independent experiment to examine the effect of systemic delivery of 21‐m in *Apoe^−^* ^/^ *^−^* mice. After 8‐week HCD and DMY treatment, those mice were randomly treated with 21‐m or NS‐m by tail vein injection for 4 weeks (two consecutives daily injection and followed by once a week for 3 weeks, 20 nmol/injection; Figure 4A). The efficiency of miR‐21 overexpression was verified by qPCR using plasma and liver samples (Figure S4A,B). Compared with *Apoe^−^* ^/^ *^−^* mice received DMY and NS‐m injection, *Apoe^−^* ^/^ *^−^* mice treatment with DMY and 21‐m had more atherosclerotic lesions in descending thoracoabdominal aorta and aortic sinus by 3.3‐fold and 1.4‐fold, respectively (Figure 4B,). Histological assessment of atherosclerotic lesions at the aortic sinus revealed a 130% increase in macrophages content by Mac‐2 staining (Figure 4D), a 210% increase in CD4‐postive T cells (Figure 4E) and a 5.5‐fold increase in SMC accumulation (Figure 4F). Moreover, systemic delivery of miR‐21 reduced circulating NO levels (Figure 4G) and increased VCAM‐1 expression in ECs at aortic sinus (Figure 4H). Furthermore, overexpression of miR‐21 in HUVECs inhibited NO production and secretion (Figure 5A‐C), increased VCAM‐1 protein and mRNA expression (Figure 5D,) and the numbers of attached monocytes to ECs (Figure 5F), and blocked the protective effects of DMY on ECs (Figure 5A‐F). The miR‐21 transfection efficiency in HUVECs was verified by qPCR (Figure 5G).

**FIGURE 6:**
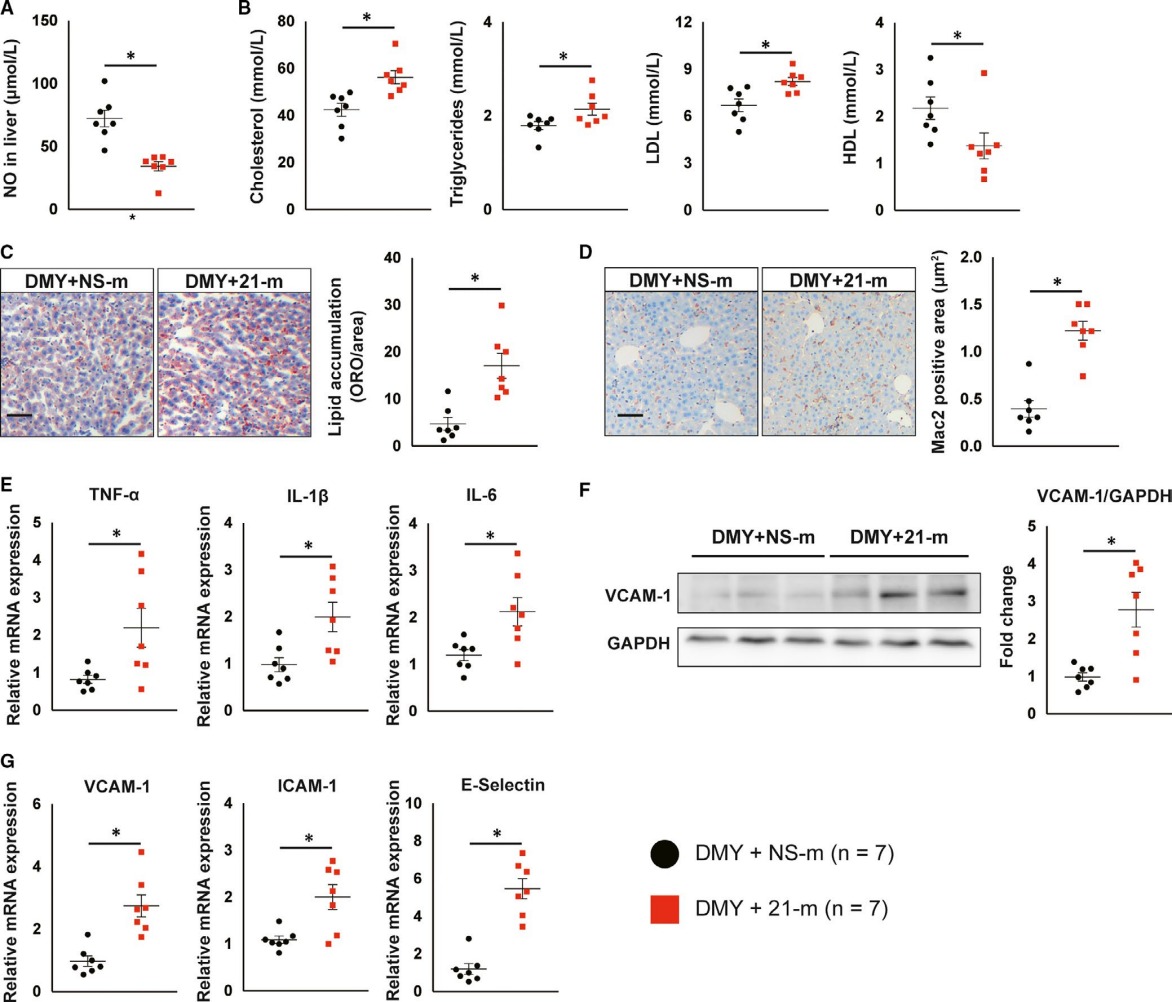
Systemic delivery of miR‐21 mimics abrogates the protective effect of dihydromyricetin (DMY) on lipid metabolism and hepatic inflammation in *Apoe^−^* ^/^ *^−^* mice. A, ELISA analysis of nitric oxide levels in livers from DMY combined with NS‐m injection or DMY combined with 21‐m injection‐treated *Apoe^−^* ^/^ *^−^* mice fed with HFD for 12 wk. B, Circulating lipid levels (Total cholesterol, Triglycerides, LDL‐C, HDL). C, Representative images and quantification show lipid accumulation in livers from DMY combined with NS‐m injection or DMY combined with 21‐m injection‐treated *Apoe^−^* ^/^ *^−^* mice fed with HFD for 12 wk. Scale: 100 μm. D, Representative images and quantification show Mac‐2–positive macrophages in livers. Scale: 100 μm. E, Real‐time qPCR analysis of indicated macrophage M1 markers in liver. The expression of genes was normalized to mouse β‐actin. F, Western blot analysis of VCAM‐1 expression in liver. G, Real‐time qPCR analysis of indicated endothelial cells activated markers in liver. The expression of genes was normalized to mouse β‐actin. Data shown are mean ± SEM (n = 7 mice per group). *P < .05

Next, we investigated whether overexpression of miR‐21 abolishes the protective effects of DMY on NO production, lipid metabolism and hepatic inflammation in liver. Compared with *Apoe^−^* ^/^ *^−^* mice received DMY and NS‐m, systemic delivery of miR‐21 mimics significantly increases miR‐21 expression (Figure S4B) and decreases NO levels in liver from mice treated with DMY (Figure 6A). Overexpression of miR‐21 increased plasma cholesterol, triglyceride and LDL levels, and decreased HDL levels (Figure 6B). In addition, systemic delivery of miR‐21 mimics significantly increased lipid accumulation in liver by Oil Red O staining (Figure 6C). Moreover, compared with NS‐m injected *Apoe^−^* ^/^ *^−^* mice, *Apoe^−^* ^/^ *^−^* mice that received miR‐21 mimics exhibited a significant increase in macrophage content indicated by Mac‐2 staining (Figure 6D), as well as macrophage M1 markers examined by qPCR (Figure 6E). Furthermore, immunoblot and qPCR assays revealed that VCAM‐1 protein and mRNA and the expression of other inflammatory genes are much higher in liver from HCD‐fed *Apoe^−^* ^/^ *^−^* mice treated with DMY and miR‐21 mimics than in DMY and NS‐m–treated *Apoe^−^* ^/^ *^−^* mice (Figure 6F,G). These data indicate that DMY increases NO generation, inhibits endothelial dysfunction, regulates lipid metabolism and inhibits hepatic inflammation by reducing miR‐21 expression. Taken together, these results (Figures 4, 5, 6) suggest miR‐21 plays an important role in the pathogenesis of atherosclerosis and DMY attenuates lipid metabolism and atherosclerotic plaque formation by decreasing miR‐21 expression.

### 3.5 Overexpression of miR‐21 abrogates DMY‐improved endothelial eNOS activation and NO production through targeting DDAH1

**FIGURE 7:**
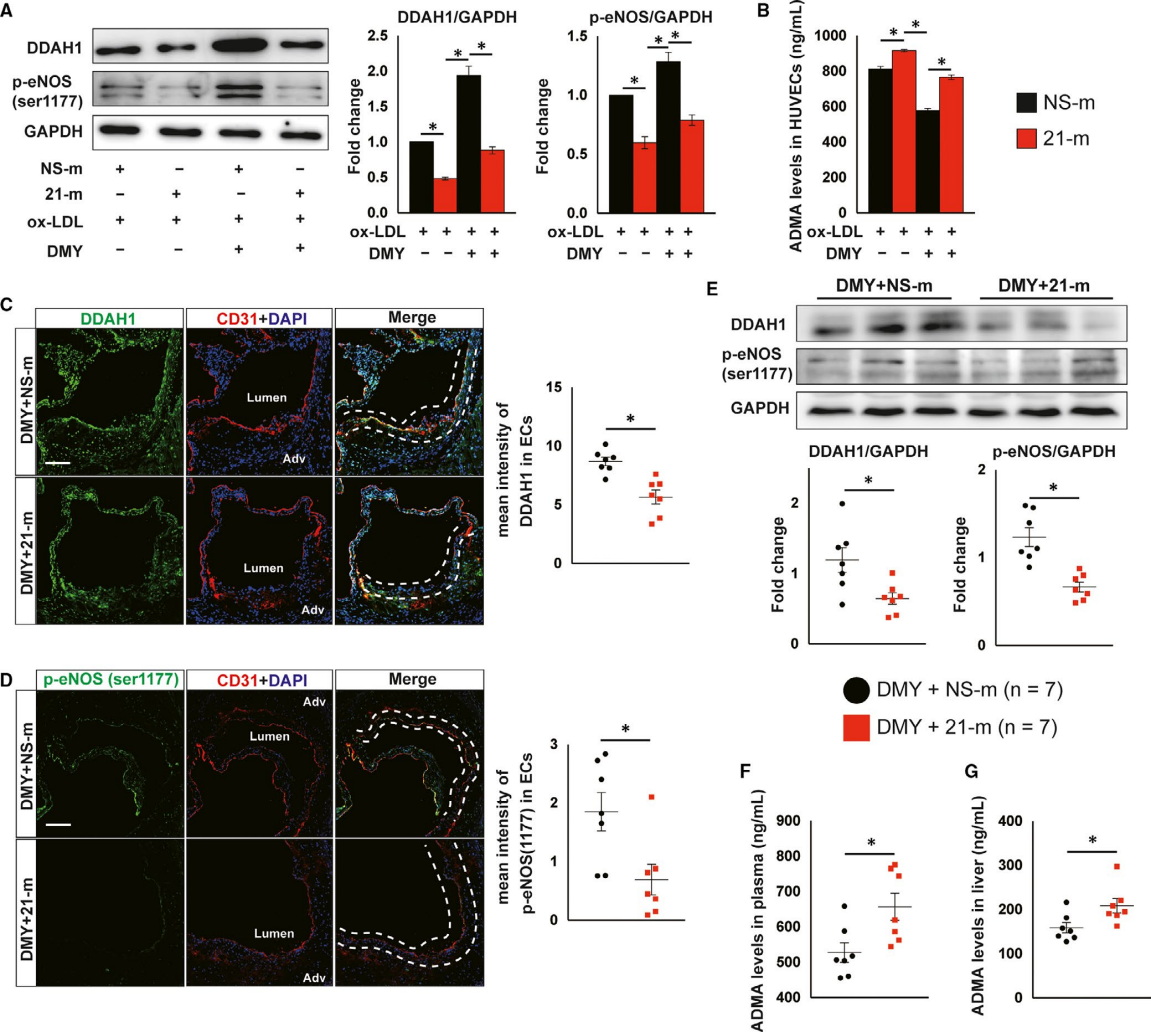
Overexpression of miR‐21 abrogates dihydromyricetin (DMY)‐increased DDAH1 expression and endothelial nitric oxide (NO) synthase phosphorylation and reduced ADMA levels. HUVECs were transfected with 100 nm agomir NC or miR‐21 agomir for 24 h and then treated with ox‐LDL (120 μg/mL) or ox‐LDL plus DMY (25 μmol/L) for 16 h and harvested for indicated experiments. A Western blot analysis of DDAH1 and p‐endothelial NO synthase (eNOS; ser1177) expression. n = 3 independent experiments. B, ELISA analysis of ADMA levels in cell lysates. n = 3 independent experiment. C, D, Representative images and quantification show DDAH1 (C) and phosphor‐eNOS (ser1177) (D) expression in endothelial cells in the aortic sinus lesions from DMY combined with NS‐m injection or DMY combined with 21‐m injection‐treated *Apoe^−^* ^/^ *^−^* mice fed with HFD for 12 wk. Frozen sections of aortic sinus were stained for anti‐DDAH1 or p‐eNOS (green), anti‐CD31 (red) and DAPI (blue). The dashed line area indicates differential DDAH1 or phosphor‐eNOS expression in endothelial cells. Scale: 50 μm. n = 7 mice per group. E, Western blot analysis of DDAH1 and p‐eNOS (ser1177) expression in liver. n = 7 mice per group. F and G, ELISA analysis of ADMA levels in plasma (F) and livers (G) from DMY combined with NS‐m injection or DMY combined with 21‐m injection‐treated *Apoe^−^* ^/^ *^−^* mice fed with HFD for 12 wk. n = 7 mice per group. Data shown are mean ± SEM. *P < .05

Our previous studies demonstrated that DDAH1 is a direct target of miR‐21 in HUVECs.^20^, ^23^, ^30^ Thus, we hypothesized that miR‐21 abrogates the protective effects of DMY on atherosclerosis by decreasing DDAH1 expression. Indeed, DMY‐increased DDAH1 protein expression in ECs in aortic sinus, liver and HUVECs (Figure S1, Figures 2 and 3). In contrast, overexpression of miR‐21 significantly decreased DMY‐induced DDAH1 protein expression in HUVECs (Figure 7A). Moreover, miR‐21 mimics abrogated DMY‐reduced intracellular ADMA levels and ‐increased phosphorylation of eNOS (Figure 7A,). These results suggest that DDAH1 expression plays an important role in DMY‐mediated eNOS‐NO activation that is controlled by miR‐21. Consistent with the in vitro data, immunofluorescence and immunoblot assay exhibited that systemic delivery of miR‐21 mimics decreases DDAH1 expression and phosphorylation of eNOS in ECs at aortic sinus compared with systemic delivery of NS‐m in DMY‐treated *Apoe^−^* ^/^ *^−^* mice (Figure 7C,), indicating that exogenous miR‐21 can abrogate DDAH1 induction and eNOS‐NO activation by DMY. Moreover, DDAH1 expression and phosphorylation of eNOS in livers from 21‐m injected *Apoe^−^* ^/^ *^−^* mice were much lower than *Apoe^−^* ^/^ *^−^* mice with NS‐m treatment while both groups of mice were treated with DMY (Figure 7E). Finally, overexpression of miR‐21 increases ADMA levels in both plasma and liver (Figure 7F,G). Collectively, these results indicate that DMY‐mediated inhibition of atherosclerosis depends on its inhibitory effect on miR‐21 expression.

## 4 DISCUSSION

Here, we show that DMY increases NO production and inhibits endothelial activation and leukocyte accumulation in liver and aortic vessel wall, thereby attenuates lipid metabolism and atherosclerotic plaque formation in *Apoe^−^* ^/^ *^−^* mice. Moreover, we demonstrate that DMY exerts these beneficial effects, at least in part, by reducing miR‐21 expression in ECs, an effect leading to the activation of DDAH1‐ADMA‐eNOS‐NO pathway. Taken together, these data demonstrate that de‐repression of DDAH1‐ADMA‐eNOS‐NO pathway due to the reduction of miR‐21 plays a critical role in the protective effects of DMY on endothelial function and atherosclerosis.

Decreased NO production in ECs caused by reduced eNOS activity contributes greatly to endothelial dysfunction, impaired vascular homeostasis and atherosclerosis.^31^, ^32^ Therefore, pharmacological approaches to maintain or restore eNOS activity in ECs are of therapeutic interest. DMY is a natural flavanonol compound that exhibited multiple pharmacological effects including anti‐oxidation.^33^ We and others recent found that DMY treatment ameliorated endothelial function in cultured HUVECs in response to TNF‐α stimulation or angiotensin II‐induced cardiomyocyte hypertrophy by increasing eNOS (ser1177) phosphorylation and NO production.^15^, ^20^ Moreover, reduced eNOS activity can also produce excessed ROS.^6^ It is possible that DMY‐mediated protective effects largely depend on eNOS‐NO pathway activation. Consistent with this hypothesis, we observed that elevated NO production and eNOS function in cultured HUVECs, endothelium and liver by DMY administration were associated with improvement of lipid metabolism and inhibition of hepatic inflammation and atherosclerosis. In support of this, NOS inhibitor L‐NAME completely abrogates those DMY‐mediated beneficial effects. While eNOS dysfunction can be caused, alone or in combination, by abnormal coupling of EC membrane receptors, insufficient supply of substrate (l‐arginine) or cofactors (tetrahydrobiopterin), or endogenous inhibitors (ADMA),^29^ this study focuses on a role of DMY in modulating DDAH1‐ADMA pathway activation. Different from prior studies,^18^, ^19^ the current study demonstrated that DMY supplementation potently increased DDAH1 expression and reduced ADMA levels, resulting in increased eNOS activity and NO production in ECs. We also detected elevated NO levels in plasma and liver, because ECs is the major source of circulating and localization of NO.^32^ However, our study did not test a direct role of DDAH1 in DMY‐mediated reduction of ADMA and eNOS activation, which will be examined in future studies.

How do DMY affects DDAH1 expression? Accumulating evidences indicate that several miRNAs play critical roles in controlling endothelial function and vascular inflammation through fine‐tuning targeted gene expression, and their dysregulation contributes to the pathogenesis of atherosclerosis.^20^, ^24^, ^25^, ^34^, ^35^, ^36^, ^37^ Our data suggest that DMY supplementation can potently decrease miR‐21 levels in plasma and liver from *Apoe^−^* ^/^ *^−^* mice and in HUVECs in response to ox‐LDL or TNF‐α stimulation.^20^ Moreover, systemic delivery of miR‐21 led to more atherosclerotic plaque and hepatic inflammation compared with NS‐m in DMY‐treated *Apoe^−^* ^/^ *^−^* mice, indicating that miR‐21 overexpression attenuates the protective effects of DMY in vivo. Indeed, clinically observed studies identified that miR‐21 expression is much higher in plasma and atherosclerotic lesions from patients compared with health control subjects and the circulating levels of miR‐21 is a sensitive predictive biomarker of coronary artery disease and future myocardial infarction.^38^, ^39^, ^40^, ^41^ Moreover, miR‐21 is significantly increased in response to multiple well‐established proatherogenic stimulus including ox‐LDL, TNF‐α and oscillatory shear stress in ECs.^20^, ^36^ Overexpression of miR‐21 potently promotes endothelial activation, dysfunction and senescence.^36^, ^37^ More importantly, miR‐21–deficient *Apoe^−^* ^/^ *^−^* mice developed less atherosclerotic plaque than miR‐21–sufficient *Apoe^−^* ^/^ *^−^* mice.^42^ We previous identified that DDAH1 is a direct target of miR‐21.^20^, ^23^, ^30^ Thus, we hypothesized that DMY modulates DDAH1 expression by decreasing miR‐21, which can enhance the activation of eNOS‐NO pathway and improve endothelial function. Our results showed that overexpression of miR‐21 inhibited eNOS‐NO pathway in both DMY‐treated *Apoe^−^* ^/^ *^−^* mice and cultured HUVECs by directly targeting DDAH1, the reduction of which increased the level of ADMA—an inhibitor of eNOS. These data revealed the molecular basis by which DMY attenuates EC dysfunction and atherosclerosis.

Limitations of the study are (a) eNOS is the dominant isoform expressed in blood vessel wall and the major source of endothelium‐derived NO,^43^ while L‐NAME can inhibit all three NOS function, not only eNOS and (b) gain‐of‐function of miR‐21 is not EC‐specific. We cannot rule out other NOS isoforms and miR‐21 in other cell types also involved in the beneficial effects of DMY on endothelial function and atherosclerosis.

## 5 CONCLUSIONS

In summary, our findings demonstrate that DMY reduces lipid burden and inhibits atherosclerosis, at least in part, by decreasing miR‐21 expression and in turn activating DDAH1‐medaited ADMA‐eNOS‐NO pathway in ECs (Figure S5). Recent clinical trials found that in patients with non‐alcoholic fatty liver disease supplemented with 600 mg DMY per day or patients with type 2 diabetes mellitus with *A grossedentata* at dose 10 g/d significantly improves glucose and lipid metabolism.^44^, ^45^ Given that the intense clinical interest in developing novel preventive and therapeutic strategies that can improve lipid metabolism and lower inflammation,^26^, ^46^ our results provide new insights into how DMY supplementation attenuates atherosclerosis, indicating that DMY may be a potential therapeutic adjuvant for treating cardiovascular diseases.

## CONFLICT OF INTEREST

The authors declare that they do not have any competing interests.

## AUTHOR CONTRIBUTIONS

S. Tan designed research; D. Yang, Z. Yang, L. Chen, D. Kuang, Y. Zou and J. Li, X. Deng, S. Luo, J. Luo, R. Li, M. Yan performed research; D. Yang, L. Chen, G. He, Q. Yuan, J. Pei and S. Tan analyzed data; D. Yang, Y. Deng, Y. Zhou, J. He and L. Chen performed statistical analysis; D. Yang and S. Tan wrote the paper. All authors reviewed and edited the manuscript and approved the final version.

## Supporting information

Supplementary methods

## Data Availability

The data that support the findings described in this study are available in the article.
