# Supplementary material for: Dihydromyricetin increases endothelial nitric oxide production and inhibits atherosclerosis through microRNA‐21 in apolipoprotein E‐deficient mice
Source: J Cell Mol Med. 2020 Apr 17;24(10):5911–25. doi: 10.1111/jcmm.15278 (PMC7214150; doi:10.1111/jcmm.15278)
Supplement: Supplementary file 1 — Supplementary methods [file JCMM-24-5911-s001.docx]

**Title: Dihydromyricetin Increases Endothelial-Nitric Oxide Production and Inhibits Atherosclerosis Through MicroRNA-21 in Apolipoprotein E-Deficient Mice**

**Author:** Dafeng Yang^1, 2*^, Zhousheng Yang^3*^, Lei Chen^1^, Dabin Kuang^4^, Yang Zou^3^, Jie Li^5^, Xu Deng^6^, Songyuan Luo^7^, Jianfang Luo^7^, Jun He^8^, Miao Yan^1^, Guixia He^9^, Yang Deng^10^, Rong Li^11^, Qiong Yuan^12^, Yangzhao Zhou^2^, Pei Jiang^13^, Shenglan Tan^1#^

^1^Department of Pharmacy, Institute of Clinic Pharmacy, Second Xiangya Hospital, Central South University, Changsha, Hunan, China

^2^Department of Cardiovascular Surgery, Second Xiangya Hospital, Central South University, Changsha, Hunan, China

^3^Department of Pharmacy, The People’s Hospital of Guangxi Zhuang Autonomous Region, Nanning, Guangxi, China

^4^Department of Pharmacy, Affiliated Changsha Hospital of Hunan Normal University, Changsha, Hunan, China

^5^Department of Geriatrics, National Key Clinic Specialty, Guangzhou First People’s Hospital, Guangzhou Medical University, Guangzhou, Guangdong, China

^6^Department of Cardiology, Third Xiangya Hospital, Central South University, Changsha, Hunan, China

^7^Department of Cardiology, Vascular Center, Guangdong Cardiovascular Institute, Guangdong Provincial Key Laboratory of Coronary Heart Disease Prevention, Guangdong Provincial People’s Hospital, Guangdong Academy of Medical Sciences, Guangzhou, Guangdong, China

^8^Department of General Surgey, Second Xiangya Hospital, Central South University, Changsha, Hunan, China

^9^School of Pharmacy, Hunan University of Chinese Medicine, Changsha, Hunan, China

^10^Department of Pharmacy, The Third Hospital of Changsha, Hunan, China

^11^The Second Affiliated Hospital of University of South China, Hengyang, Hunan, China

^12^New Drugs Innovation and Development Institute, Department of Pharmacy, College of Medicine, Wuhan University of Science and Technology, Wuhan, Hubei, China

^13^Depatment of Clinical Pharmacy and Pharmacology, Jining First People’s Hospital, Jining Medical University, Jining, Shandong, China

**Supplemental Fig. 1: DMY suppresses ox-LDL-induced EC activation by increasing NO production**

**Supplemental Fig. 2: DMY treatment decreases miR-21 expression *in vivo* and *in vitro.***

**Supplemental Fig. 3: Systemic delivery of miR-21 mimics increases miR-21 expression in mice*.***

**Supplemental Fig. 4 Systemic delivery of miR-21 mimics increases miR-21 expression in DMY-treated apolipoprotein E-deficient (*Apoe^–/–^*) mice*.***

**Supplemental Fig. 5 Depiction for proposed mechanism of DMY attenuates atherosclerosis through a miR-21-mediated DDAH1/ADMA/eNOS/NO activation in the vascular endothelium.**

**Supplemental Table S1: Primer list**


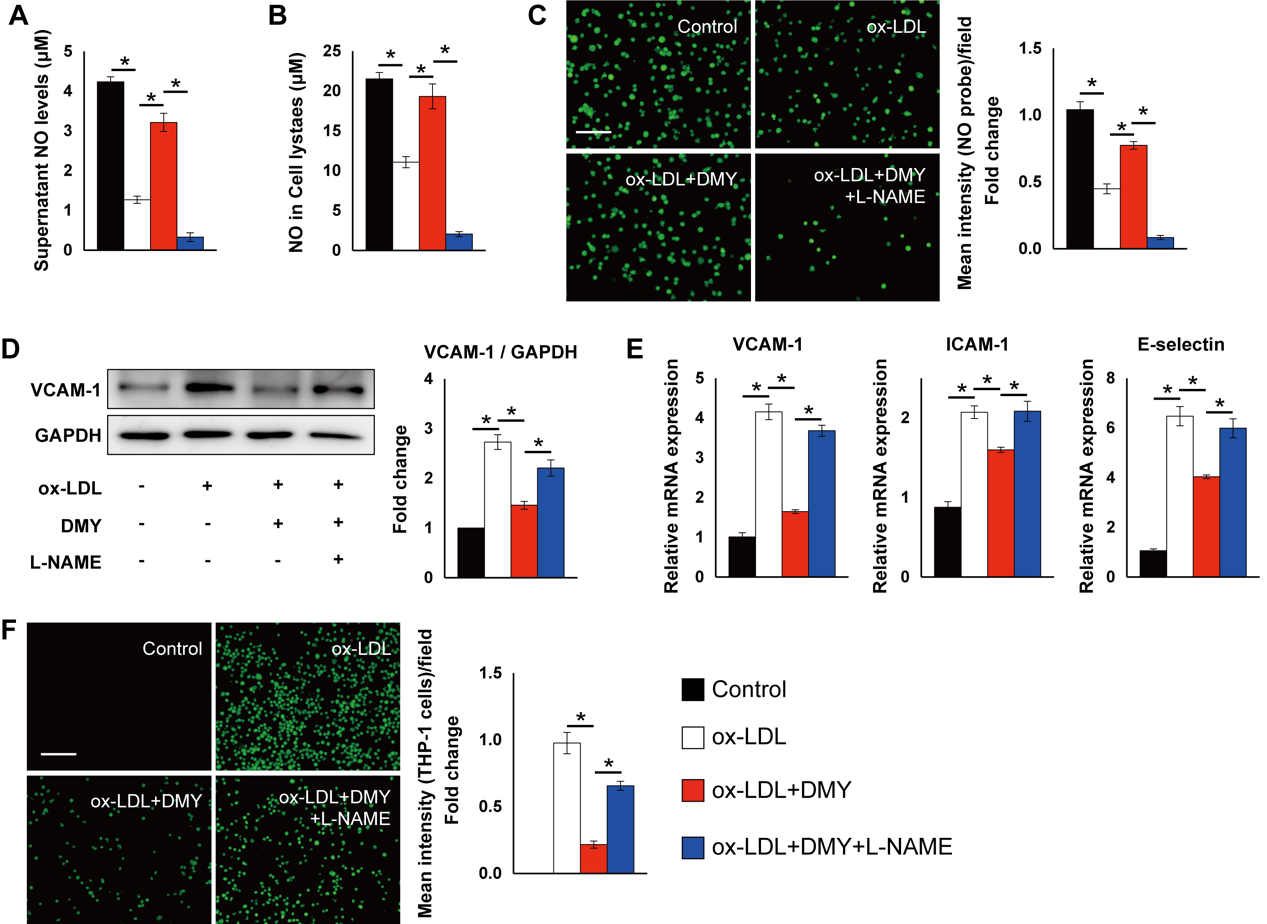


**Supplemental Fig. 1 DMY suppresses ox-LDL-induced EC activation by increasing NO production.** HUVECs were treated with ox-LDL (120 μg/ml), ox-LDL plus DMY (25 μM) or ox-LDL combined with DMY and NOS inhibitor L-NAME (25 μM) for 16 hours and harvested for indicated experiments. **A** and **B,** ELISA analysis of NO levels in supernatant (**A**) and cell lysates (**B**). n = 3 independent experiments. **C**, Fluorescent NO probe analysis of NO levels in live HUVECs. Scale: 50 μm. n = 3 independent experiments. **D**, Western blot analysis of VCAM-1 expression. n = 3 independent experiments. **E**, Real-time qPCR analysis of VCAM-1, ICAM-1 and E-selectin. Expression of VCAM-1, ICAM-1 and E-selectin were normalized to GAPDH. n = 3 independent experiments. **F**, Representative images and quantification show THP-1 cells adhering to HUVECs after treatment. Scale: 100 μm. n = 3 independent experiments. Data shown are mean ± SEM. **P* < 0.05.


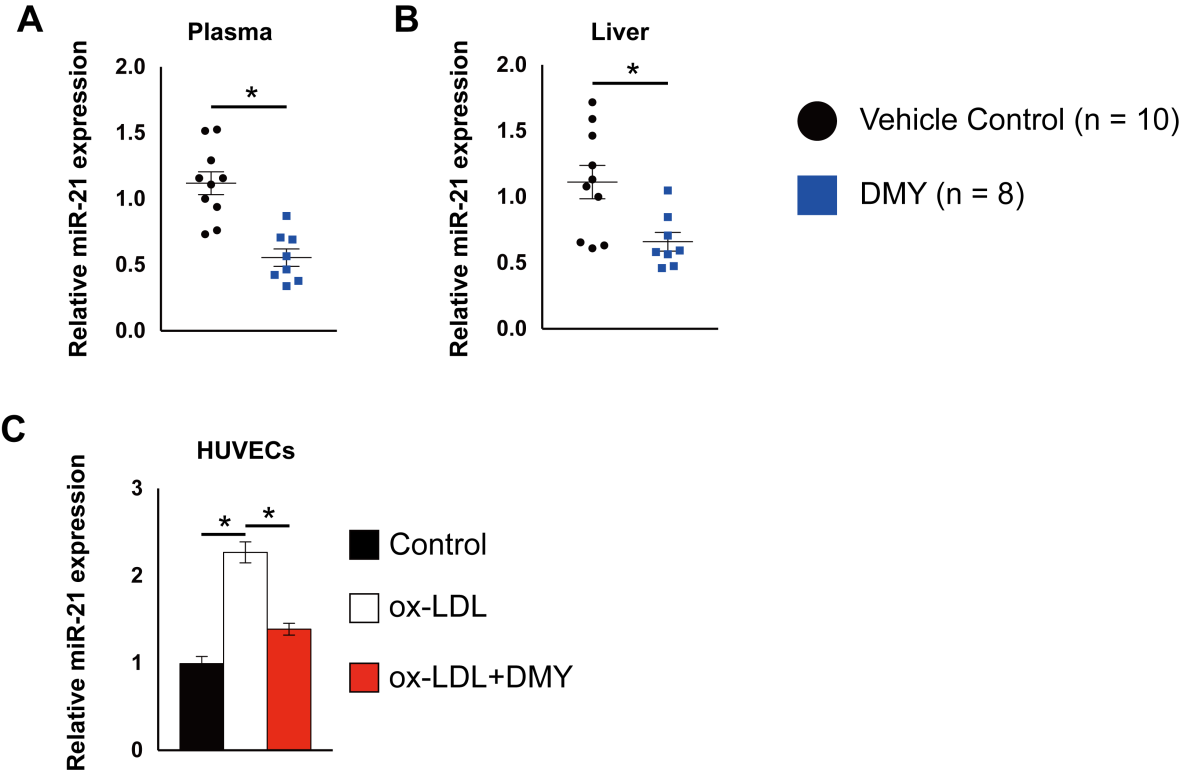


**Supplemental Fig. 2 DMY treatment decreases miR-21 expression *in vivo* and *in vitro.* A and B**, Real-time qPCR analysis of miR-21 expression in plasma (**A**) and liver (**B**) from vehicle control or DMY treated *Apoe^–/–^* mice. n = 8 - 10 mice per group. **C**, Real-time qPCR analysis of miR-21 expression in DMY-treated HUVECs in response to ox-LDL stimulation. n = 3 independent experiments. Data shown are mean ± SEM. **P* < 0.05.


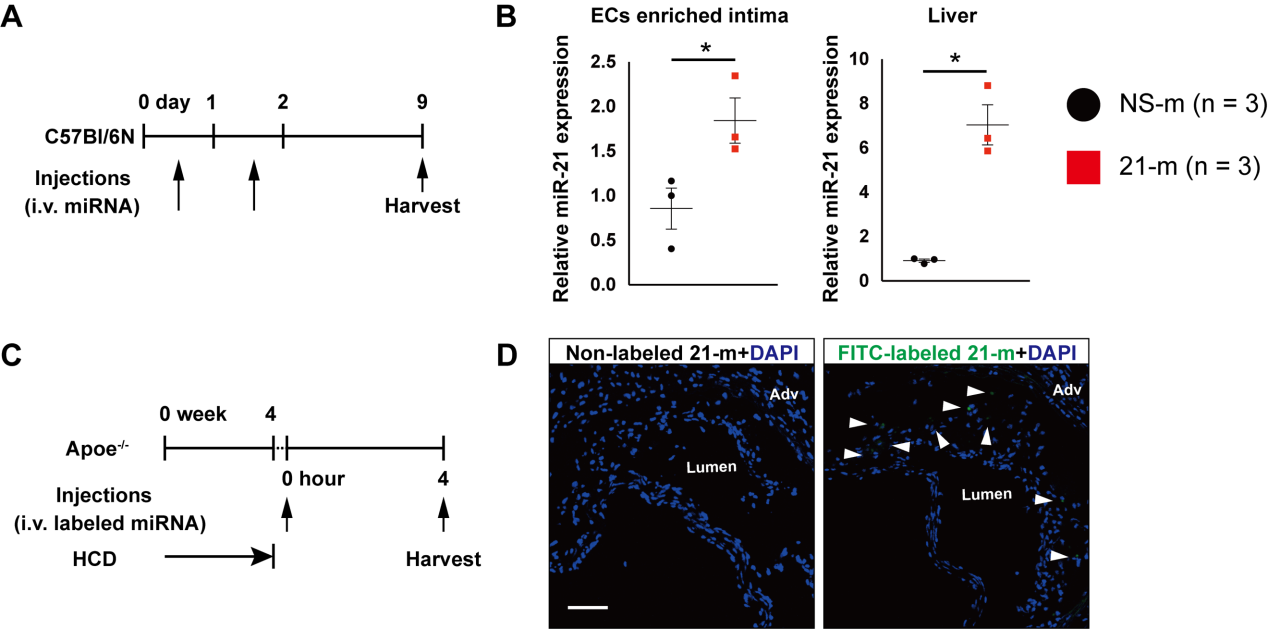


**Supplemental Fig. 3 Systemic delivery of miR-21 mimics increases miR-21 expression in mice*.* A,** Schema of experimental procedure of systemic delivery of miR-21 mimics in C57BL/6J mice. **B**, Real-time qPCR analysis of miR-21 expression in endothelial cell enriched intima and liver. n = 3 mice per group. **C,** Schema of experimental procedure of delivery FITC-labeled miR-21 mimics in apolipoprotein E-deficient (*Apoe^–/–^*) mice. D, Representative images show FITC-labeled miR-21 mimics in endothelial cells sub-endothelial area in atherosclerotic plaque in aortic sinus from *Apoe^–/–^* mice fed with HCD after 4 weeks. Frozen sections of aortic sinus were stained for 4’,6-diamidino-2-phenylindole (DAPI; blue). Arrows indicate FITC-labeled miR-21 mimics. Scale: 50 μm. Data shown are mean ± SEM. **P* < 0.05.


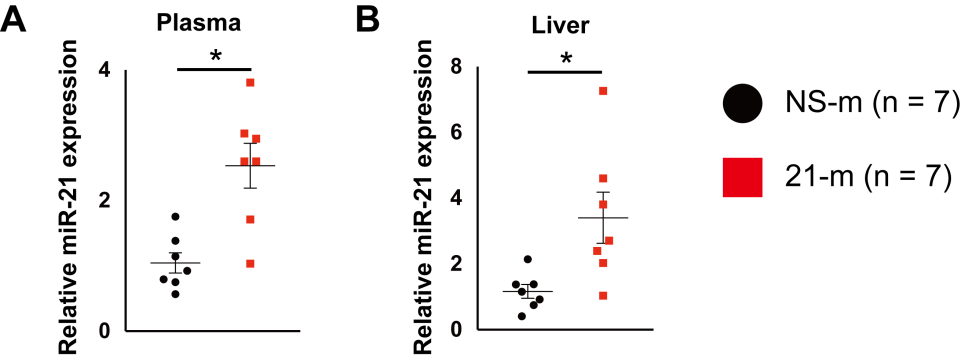


**Supplemental Fig. 4 Systemic delivery of miR-21 mimics increases miR-21 expression in DMY-treated apolipoprotein E-deficient (*Apoe^–/–^*) mice*.* A and B**, Real-time qPCR analysis of miR-21 expression in plasma (**A**) and liver (**B**) from DMY-treated *Apoe^–/–^* mice injected with non-specific mimics (NS-m) or miR-21 mimics (21-m). Data shown are mean ± SEM (n = 7 mice per group). **P* < 0.05.


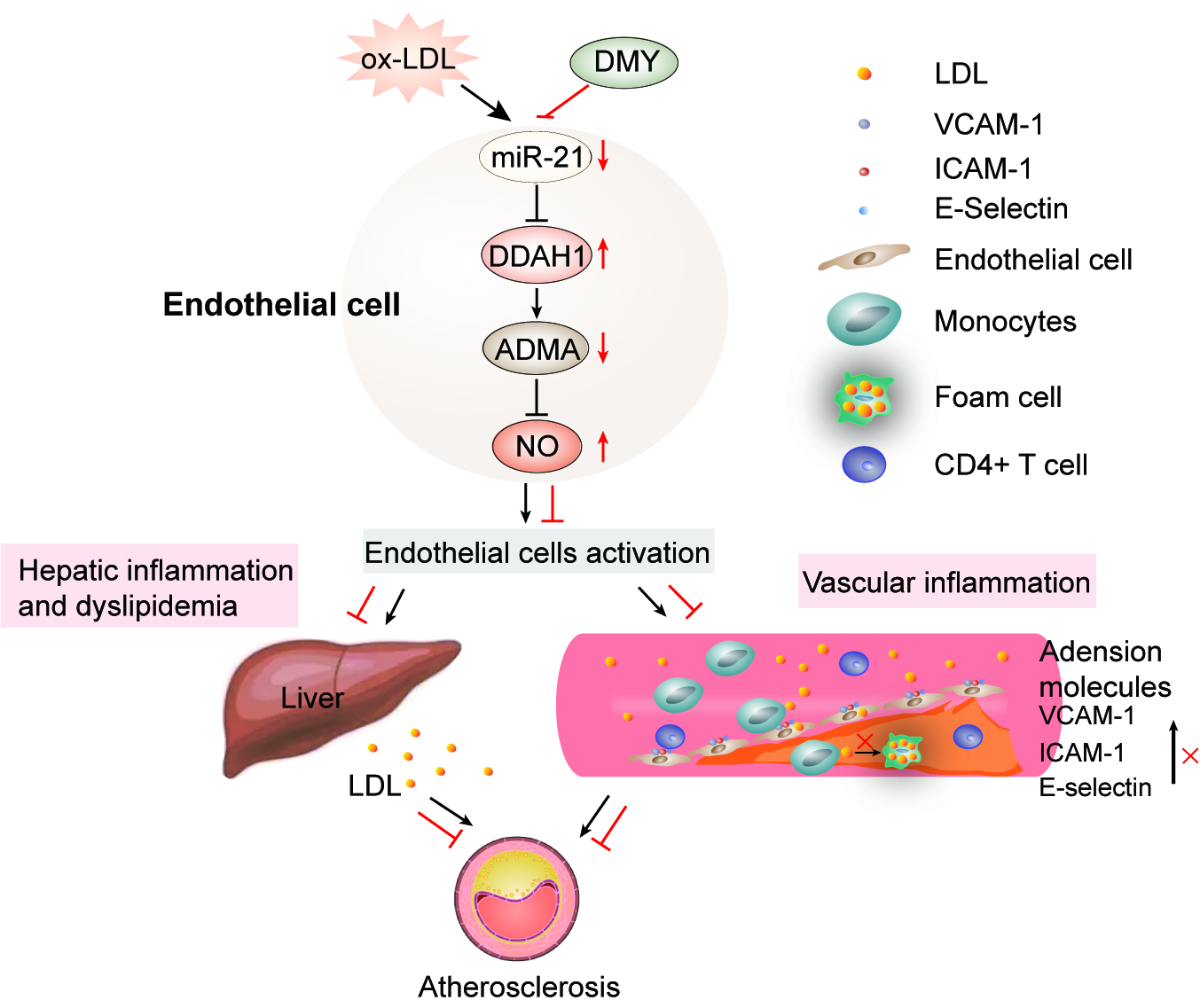


**Supplemental Fig. 5 Depiction for proposed mechanism of DMY attenuates atherosclerosis through a miR-21-mediated DDAH1/ADMA/eNOS/NO activation in the vascular endothelium.**

**Supplemental Table S1: Primer list**

| Gene name | Forward | Reverse |
| --- | --- | --- |
| Human-VCAM-1 | CCCACAGTAAGGCAGGCTGT | GCTGGAACAGGTCATGGTCA |
| Human-ICAM-1 | CGCACTCCTGGTCCTGCT | AACAACTTGGGCTGGTCACA |
| Human-E-selectin | GGGCATGTGGAATGATGAGA | CACTGAAGCCAGGGTCACAC |
| Human-GAPDH | ATGGGGAAGGTGAAGGTCG | GGGGTCATTGATGGCAACAATA |
| Mouse-VCAM-1 | CAACATGTGGCTCTGGGAAG | GCCAAACACTTGACCGTGAC |
| Mouse-ICAM-1 | GTGATGCTCAGGTATCCATCCA | AACTCTTGGCAAACATTAGGTGT |
| Mouse-E-selectin | ATGCCTCGCGCTTTCTCTC | GTAGTCCCGCTGACAGTATGC |
| Mouse-TNF-α | CCCTCACACTCAGATCATCTTCT | GCTACGACGTGGGCTACAG |
| Mouse-IL-1β | GCAACTGTTCCTGAACTCAACT | ATCTTTTGGGGTCCGTCAACT |
| Mouse-IL-6 | TAGTCCTTCCTACCCCAATTTCC | TTGGTCCTTAGCCACTCCTTC |
| Mouse-GAPDH | TGTCAAGCTCATTTCCTGGTATG | CCCTAGGCCCCTGTTAT |
